# A Comparison Between the Efficacy and Safety of Endovascular Arteriovenous Fistula Creation and Surgical Fistula Creation: A Systematic Review and Meta-Analysis

**DOI:** 10.7759/cureus.25091

**Published:** 2022-05-17

**Authors:** Yoshinosuke Shimamura, Yasutaka Kuniyoshi, Hiroshi Ueta, Takamasa Miyauchi, Hajime Yamazaki, Yasushi Tsujimoto

**Affiliations:** 1 Nephrology, Teine Keijinkai Medical Center, Sapporo, JPN; 2 Pediatrics, Tsugaru Hoken Medical Kensei Hospital, Hirosaki, JPN; 3 Anesthesiology and Critical Care, Kobe City Hospital Organization, Kobe City Medical Center General Hospital, Kobe, JPN; 4 Nephrology and Hypertension, St. Marianna University School of Medicine, Kawasaki, JPN; 5 Section of Clinical Epidemiology, Department of Community Medicine, Graduate School of Medicine, Kyoto University, Kyoto, JPN; 6 Department of Healthcare Epidemiology, Graduate School of Medicine and Public Health, Kyoto University, Kyoto, JPN

**Keywords:** technical success, medical expenditure, fistula maturation, endovascular arteriovenous fistula, chronic kidney disease (ckd)

## Abstract

An endovascular arteriovenous fistula is a recent technological advancement in hemodialysis vascular access. This systematic review and meta-analysis aimed to investigate the efficacy and safety of endovascular arteriovenous fistula (eAVF) creation compared with surgical arteriovenous fistula (sAVF) creation among patients with chronic kidney disease.

We searched Cochrane Central Register of Controlled Trials (CENTRAL), MEDLINE, EMBASE, Clinical Trials.gov, and the WHO International Clinical Trials Registry Platform until May 2021 to perform meta-analyses using random-effects models. Pre-specified primary outcomes were fistula maturation, procedure-related complications, and patient satisfaction. Secondary outcomes were procedural technical success, procedure time, all adverse events, and medical expenditure. The risk of bias in non-randomized studies of the interventions assessment tool, and the Grading of Recommendation, Assessment, Development, and Evaluation (GRADE) approach were used to assess the quality of individual studies and the body of evidence, respectively.

In seven studies including 860 patients, endovascular arteriovenous fistula creation had little to no effect on fistula maturation (odds ratio, 0.58; 95% confidence intervals, 0.05 to 6.91). Meta-analysis could not be performed for procedure-related complications and patient satisfaction due to insufficient data. For secondary outcomes, endovascular arteriovenous fistula resulted in a slight to no difference in procedural technical success (odds ratio, 0.69: 95% confidence intervals, 0.04 to 11.98) and all adverse events (odds ratio, 6.31; 95% confidence intervals, 0.64 to 62.22). Endovascular fistula creation incurred less medical expenditure than sAVF (mean difference, USD 12760; 95% confidence intervals, -19710 to -5820). Meta-analysis for procedure time was not performed because one of the studies had a critical risk of bias. All of these outcomes were of low certainty of evidence or very low certainty of evidence.

There was limited evidence for supporting endovascular arteriovenous fistula creation over conventional surgical arteriovenous fistula creation for patients with chronic kidney disease. Multicenter randomized controlled trials are needed to confirm the efficacy and safety of eAVFs in selected populations.

## Introduction and background

Chronic kidney disease (CKD) is a major health problem affecting approximately 700 million people worldwide [1]. The number of patients with CKD who receive renal replacement therapy, including hemodialysis, has increased to 2.5 million and is anticipated to reach 5.4 million worldwide by 2030 [2]. Functional vascular access is the lifeline for patients on hemodialysis [3]. The Kidney Disease Outcomes Quality Initiative guidelines [4] strongly recommend creating an arteriovenous fistula (AVF) for long-term vascular access. AVFs have been created during open surgery; however, endovascular techniques, including the Ellipsys Vascular Access System [5,6] (Avenu Medical, San Juan Capistrano, California) and the everlinQ endovascular AVF (eAVF) system [7] (TVA Medical, Austin, Texas), were approved by the Food and Drug Administration in the United States in 2018. A previous study reported the 90-day maturation rate ranged from 84 to 93 % in patients with CKD [8]. Additionally, a recent systematic review [9] demonstrated that the procedural outcomes of eAVF were similar to those of surgical AVF (sAVF); however, the prior reviews [8,9] had room for improvement with respect to the evaluation of the quality of evidence. Therefore, this systematic review and meta-analysis aimed to evaluate the available body of evidence for the efficacy and safety of eAVF compared with sAVF by applying a rigorous methodology in accordance with Preferred Reporting Items for Systematic Reviews and Meta-Analysis (PRISMA) 2020 guidelines [10] and the Cochrane Handbook [11]. We set the following research question: “Does eAVF creation have better efficacy and safety, in terms of fistula maturation, procedure-related complications, and patient satisfaction, compared with sAVF creation in patients with CKD?”

## Review

Materials and methods

Compliance With Reporting Guidelines

Using a prespecified protocol (protocols.io https://protocols.io/view/efficacy-and-safety-of-endovascular-arteriovenous-bu95nz86), we conducted a systematic review of the relevant literature in accordance with the recommendations listed in the Cochrane Handbook and in the PRISMA guidelines [10]. We confirmed that our systematic review was PRISMA-compliant through consulting the PRISMA 2020 checklist. This study was exempted from institutional review board review because of systematic review and meta-analysis of existing data.

Eligibility Criteria

We included: (ⅰ) all published articles (randomized controlled, cluster-randomized, quasi-randomized, non-randomized, and observational) that assessed the efficacy and safety of eAVF creation, (ⅱ) abstracts from conferences and letters, (ⅲ) studies of any CKD etiology, follow-up duration, publication year, and country of origin, (ⅳ) patients of any age, sex, and ethnicity, (ⅴ) studies in which patients had undergone AV graft placement, and (ⅵ) studies in terms of varying methods used for anesthesia (general or local) or in relation to varying levels of expertise and clinical experience of the operators. We excluded: (ⅰ) case reports, case series, animal and laboratory studies, ongoing studies, and literature reviews, (ⅱ) patients treated for dysfunctional hemodialysis AVFs with percutaneous transluminal angioplasty using balloons or stents, and (ⅲ) studies in which patients had undergone sAVF creation other than in the arms. Intervention was defined as any percutaneous endovascular approach to create an AVF, including the use of the Ellipsys Vascular Access System (Avenu Medical, San Juan Capistrano, California) [5], the everlinQ endovascular arteriovenous fistula system (TVA Medical, Austin, Texas) [7], and other devices. Control was defined as any surgical approach used to create or revise a forearm AVF, regardless of the method and selection of the artery and vein for anastomosis.

Outcomes of Interest

Primary outcomes were fistula maturation, procedure-related complications, and patient satisfaction. Secondary outcomes included procedural technical success, procedure time, all adverse events, and medical expenditure. Maturation was defined as AVF blood flow ≥500 ml/min and an outflow vein diameter ≥5 mm at the longest follow-up period after AVF creation, confirmed using duplex ultrasound or similar devices. The definition of procedure-related complications set by the original authors included any unintended complications directly arising from the procedure or device that occurred from the time of procedure initiation to completion. Procedural technical success was defined as the presence of blood flow in the outflow vein(s), confirmed using duplex ultrasound or similar devices. The definition of all adverse events set by the original authors included any postoperative events that required interventions on the sAVF or the eAVF. Medical expenditure was defined as the total medical financial cost including hospitalization, procedures, and materials. Definitions of patient satisfaction and procedure time established by the original authors were used.

Search Strategy and Selection of Studies

We used a set of suitable terms (details provided in Appendix Table 2-6) to search the Cochrane Central Register of Controlled Trials (CENTRAL), MEDLINE via PubMed, Excerpta Medica Database (EMBASE) via Dialogue, Clinical Trials.gov, and the WHO International Clinical Trials Registry Platform (WHO ICTRP) via their dedicated search portals until May 2021. We manually searched study reference lists, including relevant international guidelines (KDOQI Clinical Practice Guideline for Vascular Access: 2019 updates) [4] as well as the reference lists of eligible studies and articles citing eligible studies. We asked the authors of the original studies for unpublished or additional data if the database entry for a candidate study did not contain the necessary information. Two of four reviewers (YS, HU, TM, and HY) independently screened the titles and abstracts of each study identified in the search to determine whether the inclusion criteria were met. Three independent reviewers (YS, HU, and TM) performed full-text reviews to assess the eligibility of each candidate study. Disagreements were resolved through discussion among the three reviewers and, if an agreement was not met, a fourth reviewer acted as an arbiter (YT or YK).

Data Abstraction and Quality Assessment

Three reviewers (YS, HU, and TM) independently extracted data from the included studies using a standardized data collection form. Disagreements regarding data extraction were resolved through discussion. When necessary, we contacted the authors of studies that did not provide sufficient information. Three reviewers (YS, HU, and TM) independently evaluated the risk of bias using the ROBINS-I tool [12]. Differences in opinion regarding the assessment of the risk of bias were resolved through discussion among the three reviewers, occasionally with arbitration by a fourth reviewer (YT or YK).

Data Analysis

All analyses were conducted using Cochrane Review Manager software (RevMan v.5.4; Cochrane Collaboration, Copenhagen, Denmark). For the dichotomous outcomes of maturation, procedure-related complications, procedural technical success, and all adverse events, pooled odds ratios (ORs) with 95% confidence intervals (CIs) are provided. If adjusted ORs and 95% CIs of the outcomes were not available and if the risk ratios (RRs) had been reported in the primary studies, we used a method reported by Zhang et al. [13] to transform RRs into ORs. If only hazard ratios (HRs) were reported, we contacted the authors to confirm the ORs and/or RRs. If ORs were not available after querying the authors, we integrated them with ORs and HRs. For continuous outcomes, including patient satisfaction, procedure time, and medical expenditure, the standardized mean differences or the mean differences with 95% CIs were calculated as recommended in the Cochrane Handbook [11]. We used random-effects models for all analyses.

We evaluated statistical heterogeneity through a visual inspection of forest plots and through calculating the I2 statistic, which interpreted the values as follows: 0% to 40%, negligible heterogeneity; 30 to 60%, mild-to-moderate heterogeneity; 50% to 90%, moderate-to-substantial heterogeneity; and 75% to 100%, considerable heterogeneity. We investigated the underlying reasons for heterogeneity and conducted a chi-squared test, and a p-value <0.10 was considered to indicate statistical significance. We investigated reporting bias by checking ClinicalTrials.gov and the WHO ICTRP to identify trials that had been completed but not published at the time of the review.

Concerning the primary outcomes, the following pre-specified sensitivity analyses were conducted: (ⅰ) exclusion of studies using imputed statistics, and (ii) inclusion of studies with complete case data. We also performed pre-specified subgroup analyses according to the etiology of CKD (diabetes-related nephropathy versus non-diabetes-related nephropathy), type of endovascular device (Ellipsys Vascular Access System versus everlinQ endovascular AVF system), type of control (the use of native arteries and veins versus the use of arteriovenous grafts), type of study (randomized controlled trials versus non-randomized controlled trials), and the number of AVF procedures (the first AVF creation versus the revascularization of AVFs). We created a summary of findings table that included an overall grading of the certainty of the evidence for the following outcomes: maturation, procedural technical success, procedure time, all adverse events, and medical expenditure. Two reviewers (YS and YT) evaluated the certainty of evidence based on the Grading of Recommendation, Assessment, Development, and Evaluation (GRADE) approach [14]. Disagreements between these two reviewers were discussed and, if a consensus was not met, a third reviewer (YK) acted as an arbiter.

Patient and Public Involvement

No patients or members of the public were involved in this meta-analysis.

Results

Study Characteristics

**Figure 1:**
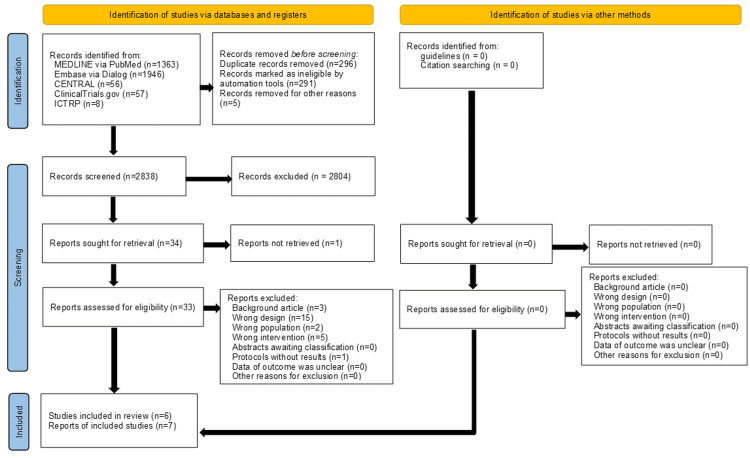
Preferred Reporting Items for Systematic reviews and Meta-Analyses flow diagram

After removing duplicates, we identified 2838 studies during the search conducted until May 2021. We identified eight studies that fulfilled all eligibility criteria and we included them in the qualitative synthesis [15-22]. After excluding one ongoing study (protocol without results) [22], a total pooled sample of 860 patients (eAVF group, n = 429 patients; sAVF group, n = 431) from seven studies (six single-center observational studies and one population-based study) was included in the quantitative synthesis (Figure 1).

Table 1 shows the characteristics of included studies. Three studies were performed in the United States, and the other studies were performed in Germany, the United Kingdom, and France. The mean or median age of patients in these analyzed studies ranged from 56.7 to 67.0 years. Four studies [15,16,20,21] had received research funding from manufacturers.

**Table 1:** Characteristics of included studies BB, brachiobasilic arteriovenous fistula; BC, brachiocephalic arteriovenous fistula; BMI, body mass index; eAVF, endovascular arteriovenous fistula; M, multi-ethnicity; NA, not available; P, population-based study; PY, person-year; RC, radiocephalic arteriovenous fistula; UK, United Kingdom; US, United States; USD, United States dollars; sAVF, surgical arteriovenous fistula; S, single-center study. ^a ^The procedure was performed by a single operator; ^b ^Median (interquartile range); ^c ^Mean ± standard deviation. Shahverdyan et al. [15]; Yang et al. [16]; Arnold et al. [17]; Inston et al. [18]; Osofsky et al. [19]; Harika et al. [20]; Hull et al. [21]

Author	Year	Country	Settings	Source of funding	Intervention (Device)	Comparator (Technique)	Sample size (n)	Age (years)	BMI (kg/m^2^)	Ethnicity	Previous failed AVF	Follow-up (days)	Measured outcomes
Shahverdyan et al.	2021	Germany	S	None	eAVF ^a^ (Ellipsys)	sAVF (Gracz)	158	eAVF: 66.0 (28.0-86.2) ^a^ sAVF: 67.9 (33.2-87.7) ^a^	eAVF: 28.7 (16.5-50.2) ^b^ sAVF: 26.2 (16.5-45.1) ^b^	NA	0	355.7 (1-1061) ^b^	Maturation
Yang et al.	2017	The U.S.	P	TVA Medical Inc.	eAVF (everlinQ)	sAVF (NA)	120	eAVF: 60.0 ± 15.3^ c^ sAVF: 61.1 ± 13.6^ c^	eAVF: 27.9 ± 6.1^ c^ sAVF: NA	M	eAVF: 32 % sAVF: 25 %	NA	Medical expenditure
Arnold et al.	2018	The U.S.	S	TVA Medical Inc.	eAVF (everlinQ)	sAVF (NA)	120	eAVF: 57.0 sAVF: 64.8	eAVF: 27.1 ± 6.3^ c^ sAVF: 29.9 ± 8.4^ c^	M	eAVF: 32 % sAVF: NA	NA	Medical expenditure
Inston et al.	2020	The U.K.	S	None	eAVF (WavelinQ)	sAVF (RC)	70	eAVF: 57 ± 15^ c^ sAVF: 54 ± 17^ c^	NA	M	0	eAVF: 497 ± 187^ c^ sAVF: 468 ± 148^ c^	Maturation Procedural-technical success All adverse events
Osofsky et al.	2021	The U.S.	S	None	eAVF (Ellipsys)	sAVF (RC)	86	eAVF: 56.7 ± 22.6^ c^ sAVF: 62.5 ± 13.2^ c^	eAVF: 30.5 ± 6.7^ c^ sAVF: 28.8 ± 6.8^ c^	NA	eAVF: 25 sAVF: 27	eAVF: 6.1 ± 4.0 months ^c^ SAVF: 2.7 ± 2.6 months ^c^	Maturation Procedural-technical success Procedure time All adverse events
Harika et al.	2021	France	S	Avenu Medical	eAVF ^a^ (Ellipsys)	sAVF (RC, BC, BB)	214	eAVF: 63.6 ± 15.14^ c^ sAVF: 63.5 ± 15.69^ c^	eAVF: 27.2 ± 5.78^ c^ sAVF: 26.8 ± 5.95^ c^	NA	NA	NA	Maturation
Hull et al.	2020	The U.S.	S	Avenu Medical	eAVF (Ellipsys)	sAVF (NA)	130	eAVF: 64 ± 14^ c^ sAVF: NA	eAVF: 30.7 ± 9.0^ c^ sAVF: NA	M	0	282 (103-385)^ c^	Procedure time

Primary Outcomes

**Figure 2:**
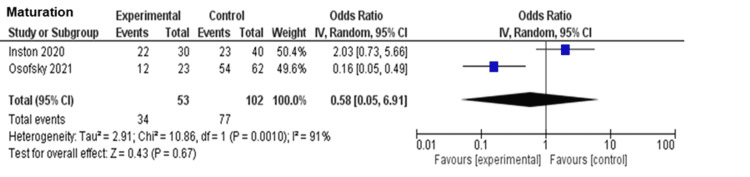
Forest plot for the primary outcome

**Figure 3:**
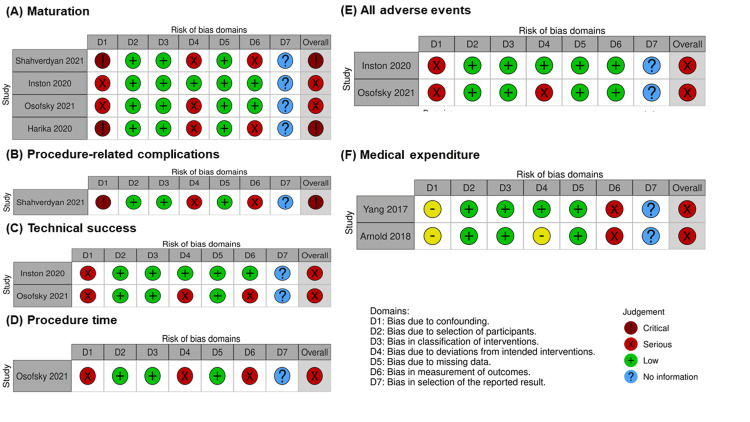
Risk of bias in non-randomized studies of interventions

**Figure 4:**
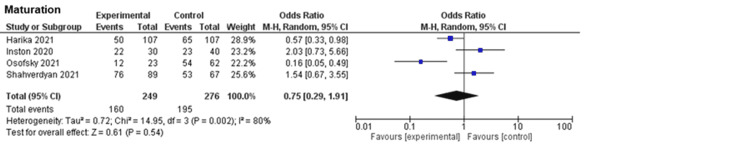
Post-hoc sensitivity analysis for maturation

Table 2 summarizes the findings of this review. Fistula maturation was measured in four studies [15,18-20]. Of these, two studies [15,20] were excluded from the meta-analysis due to the critical risk of bias. The creation of eAVF had little to no effect on maturation, but the evidence was very uncertain (OR 0.58, 95% CI 0.05 to 6.91; I2 = 91%, in two studies [18,19]; n = 155) with very low certainty of evidence (Figure 2 and Figure 3A). Although one study [15] measured procedure-related complications, it was not suitable for quantitative analysis due to the critical risk of bias. None of the studies had measured patient satisfaction. We could not perform all prespecified sensitivity analyses because no study used imputed statistics or included complete case data. However, posthoc sensitivity analysis for fistula maturation, including studies with a critical risk of bias, had similar results (OR 0.75, 95% CI 0.29 to 1.91; I2 = 80%, in four studies [15,18-20]; n = 525) (Figure 4). We did not perform prespecified subgroup analyses for primary outcomes because of insufficient data.

**Table 2:** Summary of findings GRADE Working Group grades of evidence. High certainty: we are very confident that the true effect lies close to that of the estimate of the effect. Moderate certainty: we are moderately confident in the effect estimate: the true effect is likely to be close to the estimate of the effect, but there is a possibility that it is substantially different. Low certainty: our confidence in the effect estimate is limited: the true effect may be substantially different from the estimate of the effect. Very low certainty: we have very little confidence in the effect estimate: the true effect is likely to be substantially different from the estimate of effect.

Endovascular arteriovenous fistula creation compared with surgical arteriovenous fistula creation for the vascular access in patients with end-stage kidney disease						
Outcomes	Anticipated absolute effects^*^ (95% CI)		Relative effect (95% CI)	№ of participants (studies)	Certainty of the evidence (GRADE)	Comments
	The risk with surgically created fistulas	The risk with endovascular arteriovenous fistula creation				
Maturation	755 per 1,000	641 per 1,000 (133 to 955)	OR 0.58 (0.05 to 6.91)	155 (2 observational studies)	⨁◯◯◯ Very low ^a,b,c^	^a ^serious risk of bias by ROBINS-I; ^b ^odds ratios ranged from 0.05 to 6.91 and I^2^ was 91%; ^c^ only two small studies were included.
Procedural technical success	970 per 1,000	957 per 1,000 (564 to 997)	OR 0.69 (0.04 to 11.98)	153 (2 observational studies)	⨁◯◯◯ Very low ^a,c,d^	^a^ serious risk of bias by ROBINS-I; ^c^ only two small studies were included; ^d^ odds ratios ranged from 0.04 to 11.98 and I^2^ was 53%.
Procedure time	The mean procedure time was 56 minutes	mean 4 minutes higher (13.17 lower to 21.17 higher)	Not available	86 (1 observational study)	⨁⨁◯◯ Low ^a,e^	^a ^serious risk of bias by ROBINS-I; ^e ^only one small study was included.
All adverse events	333 per 1,000	759 per 1,000 (242 to 969)	OR 6.31 (0.64 to 62.22)	155 (2 observational studies)	⨁◯◯◯ Very low ^a,c,f^	^a ^serious risk of bias by ROBINS-I; ^c^ only two small studies were included; ^f^ odds ratios ranged from 0.64 to 62.22 and I^2^ was 84%.
Medical expenditure (U.S. dollars)	The mean medical expenditure (U.S. dollars) was 13778 U.S. dollars	MD 12760 U.S. dollars lower (19710 lower to 5820 lower)	Not available	120 (2 observational studies)	⨁⨁◯◯ Low ^a,c^	^a ^serious risk of bias by ROBINS-I; ^c^ only two small studies were included.

Secondary Outcomes

**Figure 5:**
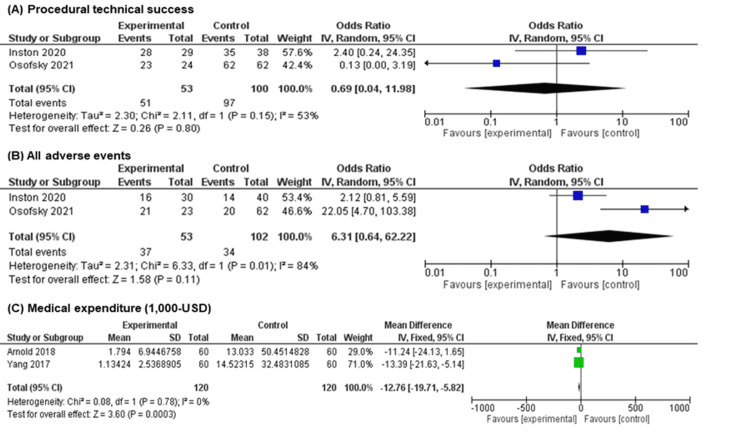
Forest plot for the secondary outcomes

Figure 5 shows the forest plots for the secondary outcomes. The evidence was very unclear concerning the effect of eAVF creation on procedural technical success (OR 0.69, 95% CI 0.04 to 11.98; I2 = 53%, in two studies [18,19]; n = 153) with a very low certainty of evidence, and in terms of all adverse events (OR 6.31, 95% CI 0.64 to 62.22; I2 = 84%, in two studies [18,19]; n = 155) with a very low certainty of evidence. In contrast, eAVF resulted in a large reduction in medical expenditure (mean difference, $ 12,760, 95% CI, -19,710 to -5,820; I2 = 0%, in two studies [16,17]; n = 120), but there was a low certainty of evidence. Procedure time was measured in two studies [19,21]; however, a quantitative analysis of this outcome was not performed because one of the studies [21] had a critical risk of bias. Details concerning the risk of bias for secondary outcomes are provided in Figures 3B-3F above.

Discussion

Our review included seven studies that involved 860 patients with CKD. Our findings indicated that evidence is very uncertain about the effects of eAVF creation on the maturation rate compared with sAVF creation. The certainty of the evidence was downgraded to very low levels because of the inclusion of retrospective cohort studies, a serious risk of bias, and imprecise estimates. We were not able to confirm the effect of eAVF on procedure-related complications due to limited data and a critical risk of bias. Moreover, no study reported on patient satisfaction. Additionally, it remains uncertain whether eAVF increases procedural technical success or reduces all adverse events because the results were based on very low certainty of evidence. However, our findings indicated that eAVF reduced medical expenditures related to patient care, which may suggest a cost-effective alternative to sAVF.

The findings of this systematic review and meta-analysis suggest that, based on current evidence, eAVF cannot be confirmed as more efficacious or safer than sAVF. This conclusion is in contrast with conclusions drawn in a recent systematic review [9] that claimed that eAVF creation was a safe alternative with outcomes comparable to those for surgery. One reason for this discrepancy may be related to a difference in the number of included studies. While the prior study [9] selected five studies, we selected seven studies, suggesting that a more comprehensive literature search might be conducted in this review. Another reason may be related to a difference in the interpretation of results. According to GRADE guidelines [14], review authors are recommended to downgrade the certainty of the evidence if CIs are wide. In this respect, we graded the certainty of evidence accordingly and provided a rationale for grading based on the GRADE approach [14].

Our systematic review comprehensively summarized the available evidence on the efficacy and safety of eAVF compared with sAVF. Another meta-analysis [8] evaluating single-arm studies of eAVF showed a similar technical success rate of 97.5% but a higher maturation rate of 89.27% than found in our review (ranging from 52% to 73%). In addition to differences in study design, facility specialization may help explain these discordant results. One study [19] was conducted in the early phase of an eAVF program at an institution. While there is currently no conclusive evidence concerning the effect of eAVF on maturation, changes to clinical practice may be advised if further relevant information from an ongoing trial (NCT04404985) [22] becomes available.

Our finding that medical costs were less with eAVF deserves attention because previous meta-analyses [8,9] have not addressed this issue. Although the certainty of the evidence was low, our data showed that eAVF saved approximately USD 12,760 compared with sAVF. Because the relative effect of eAVF in relation to sAVF remains to be elucidated, more data are needed regarding medical expenditure for eAVF to evaluate its cost-effectiveness. This information is important for patients and medical professionals to guide decision-making in clinical settings. It is also essential for policymakers to determine whether medical resources should be allocated to eAVF or other vascular access methods.

The results of this meta-analysis may be applicable to patients with excess weight because the populations in our review [16-21] included participants with excess body weight, which makes AVF creation be challenging. Similarly, our results can also be applicable to diverse ethnic groups because patients of African and Asian ethnicity were included in four studies [16-18,21]. Notably, the results of the present study need to be interpreted with caution because they had a low certainty of evidence.

Our results have several clinical and research implications. First, the eAVF was not found to be an alternative to sAVF because it remains unclear whether creating an eAVF is more beneficial or harmful than an sAVF. To determine if eAVFs can be used routinely, high-quality clinical trials aimed at incorporating eAVFs into routine clinical practice for patients with CKD are warranted. Second, patient satisfaction should be measured as a patient-oriented outcome because patients’ opinions offer valuable insight into this topic and contribute to high-quality patient care. For instance, the ease of cannulation between eAVF and sAVF could be used as a measurement item to assess patient satisfaction.

Limitations and strengths

This systematic review had several limitations. First, we excluded studies [15,20] with a critical risk of bias from the main analysis, resulting in a smaller number of studies included in our meta-analysis. However, we included those with a critical risk of bias in the posthoc sensitivity analysis and confirmed that the results were similar to those of the main analysis. Second, we had a suboptimal number of participants to undertake quantitative data syntheses [23]. Moreover, the small number of data observations limited our ability to conduct robust statistical estimates of outcomes and prespecified subgroup analyses. Third, potential publication bias was not readily discernable in the funnel plot because we included fewer than 10 studies [14]. Additionally, we did not find any conference abstract in the literature search, and this may be unlikely to affect our estimates. Fourth, most of the included studies were single-center observational studies, which have inherent potential confounding variables.

The strengths of our review were that it involved a comprehensive search for evidence in accordance with the PRISMA statement [10] and the employment of the GRADE approach [14] to assess the certainty of evidence. In addition, three investigators independently performed data extraction and analyses to minimize the risk of errors in determining study eligibility, as well as for data extraction, risk of bias assessment, and data synthesis. Fifth, adverse events were measured in different ways and the definition varied among studies, which precluded comparisons of results between studies and meta-analyses. A standardized measurement of outcomes is recommended because it is likely to contribute to improving the quality of future trials on this topic as well as mitigating heterogeneity across studies.

## Conclusions

In conclusion, this review showed that evidence for supporting eAVF creation over conventional sAVF was very limited among patients with CKD requiring hemodialysis. We strongly recommend further multicenter, prospective, randomized controlled trials to determine the efficacy and safety of eAVFs in this population.

## Appendices

**Table 3:** MEDLINE (PubMed) search strategy

	MEDLINE (PubMed) search strategy
#1	Kidney Diseases[mh]
#2	Renal Dialysis[mh]
#3	ESRD[tiab]
#4	end stage renal disease[tiab]
#5	ESKD[tiab]
#6	end stage kidney disease[tiab]
#7	CKD[tiab]
#8	chronic kidney disease[tiab]
#9	chronic renal failure[tiab]
#10	#1 OR #2 OR #3 OR #4 OR #5 OR #6 OR #7 OR #8 OR #9
#11	Arteriovenous Shunt, Surgical[mh]
#12	Endovascular Procedures[mh]
#13	#11 AND #12
#14	intravascular procedures[tiab]
#15	intravascular techniques[tiab]
#16	percutaneous arteriovenous fistula[tiab]
#17	Ellipsys[tiab]
#18	everlin Q[tiab]
#19	hemodialysis shunt[tiab]
#20	#13 OR #14 OR #15 OR #16 OR #17 OR #18 OR #19
#21	#9 AND #20

**Table 4:** CENTRAL (Cochrane Library) search strategy CENTRAL, Cochrane Central Register of Controlled Trials

	CENTRAL (Cochrane Library) search strategy
#1	[mh "Renal Dialysis"]
#2	[mh "Kidney Diseases"]
#3	ESRD:ti,ab
#4	"end stage renal disease":ti,ab
#5	ESKD:ti,ab
#6	"end stage kidney disease":ti,ab
#7	CKD:ti,ab
#8	"chronic kidney disease":ti,ab
#9	"chronic renal failure":ti,ab
#10	#1 OR #2 OR #3 OR #4 OR #5 OR #6 OR #7 OR #8 OR #9
#11	[mh "Arteriovenous Shunt, Surgical"]
#12	[mh "Endovascular Procedures"]
#13	#11 AND #12
#14	"intravascular procedures":ti,ab
#15	"intravascular techniques":ti,ab
#16	"percutaneous arteriovenous fistula":ti,ab
#17	Ellipsys:ti,ab
#18	everlinQ:ti,ab
#19	"hemodialysis shunt":ti,ab
#20	#13 OR #14 OR #15 OR #16 OR #17 OR #18 OR #19
#21	#9 AND #20

**Table 5:** EMBASE (Dialog) search strategy

	EMBASE (Dialog) search strategy
S1	"EMB.EXACT.EXPLODE("kidney disease")
S2	"EMB.EXACT.EXPLODE("hemodialysis")
S3	ab(ESRD) OR ti(ESRD)
S4	ab(end stage renal disease) OR ti(end stage renal disease)
S5	ab(ESKD) OR ti(ESKD)
S6	ab(end stage kidney disease) OR ti(end stage kidney disease)
S7	ab(CKD) OR ti(CKD)
S8	ab(chronic kidney disease) OR ti(chronic kidney disease)
S9	ab(chronic renal failure) OR ti(chronic renal failure)
S10	S1 OR S2 OR S3 OR S4 OR S5 OR S6 OR S7 OR S8
S11	"EMB.EXACT.EXPLODE("arteriovenous shunt")
S12	"EMB.EXACT.EXPLODE("endovascular surgery")
S13	S11 AND S12
S14	ab(intravascular procedures) OR ti(intravascular procedures)
S15	ab(intravascular techniques) OR ti(intravascular techniques)
S16	ab(percutaneous arteriovenous fistula) OR ti(percutaneous arteriovenous fistula)
S17	ab(Ellipsys) OR ti(Ellipsys)
S18	ab(everlin Q) OR ti(everlin Q)
S19	ab(hemodialysis shunt) OR ti(hemodialysis shunt)
S20	S13 OR S14 OR S15 OR S16 OR S17 OR S18 OR S19
S21	S9 AND S20

**Table 6:** ICTRP search strategy ICTRP, International Clinical Trials Registry Platform

ICTRP search strategy
#1 Conditions: (chronic kidney disease and renal dialysis) #2 Intervention: (percutaneous arteriovenous fistula, Ellipsys, everlin Q) #3 #1 AND #2 Recruitment status is ALL.

**Table 7:** ClinicalTrials.gov search strategy

ClinicalTrials.gov search strategy
#1 Conditions: (chronic kidney disease and renal dialysis) #2 Intervention: (percutaneous arteriovenous fistula, Ellipsys, everlin Q) #3 #1 AND #2 Recruitment status is ALL.
